# PROMISES AND CHALLENGES OF IMPLEMENTING TELEHEALTH IN LTC FACILITIES: LESSONS FROM A MULTISITE PILOT PROJECT

**DOI:** 10.1093/geroni/igad104.1146

**Published:** 2023-12-21

**Authors:** Mengzhao Yan, Yujun Zhu, Kathleen Wilber

**Affiliations:** University of Southern California, Los Angeles, California, United States; University of Southern California, Los Angeles, California, United States; University of Southern California, Los Angeles, California, United States

## Abstract

In response to the COVID-19 pandemic, the demand for telehealth implementation has expanded in long-term care (LTC) facilities. To better understand the promises and challenges of implementing telehealth, we evaluated a pilot project in which five LTC facilities in California implemented telehealth from September 2021 to May 2022. Employing a mix-method approach, we used a diverse set of evaluation tools (i.e., documenting 37 telehealth visits, holding 8 monthly learning sessions, conducting 36 check-in interviews, and tracking monthly COVID-19 cases) to examine how telehealth was adopted, used, and perceived among residents, administrators, and health practitioners. From a descriptive analysis of quantitative data and a thematic analysis of qualitative data, we found that telehealth demonstrated the capability to treat a variety of conditions; prevented nearly 20% of transfers to emergency departments; residents indicated a high degree of comfort with telehealth; and healthcare practitioners perceived that telehealth could improve care delivery. In addition to benefits, several barriers were identified including initial concerns about the quality of visits, required upfront costs, time constraints, difficulties in coordinating different platforms in the healthcare system, uncertainty about payment parity with in-person care, and reduced staffing related to COVID-19. Based on our evaluation, we propose six strategies to help implement telehealth in LTC facilities: recognize sources of resistance to change; recognize the need for initial investment; understand and address contextual issues; prepare and pave the way for staff buy-in; identify a champion; and ensure sufficient and ongoing training and support.

